# Quantitative assessment and localization of the hollowing of the temple after craniectomy and cranioplasty–The frontozygomatic shadow

**DOI:** 10.1371/journal.pone.0258776

**Published:** 2021-10-19

**Authors:** Michael Kosterhon, Eva Ruegg, Malte Ottenhausen, Anne Kühn, Florian Ringel, Max Jägersberg

**Affiliations:** 1 Department of Neurosurgery, University Medical Center of the Johannes Gutenberg-University Mainz, Mainz, Germany; 2 Clinic of Plastic, Reconstructive and Esthetic Surgery, Hirslanden Private Hospital Group, Lucerne, Switzerland; I.R.C.C.S. San Raffaele Scientific Institute, Vita-Salute San Raffaele University, ITALY

## Abstract

**Background:**

After cranioplasty, in many cases a not negligible soft tissue defect remains in the temporozygomatical area, also referred to as a hollowing defect of the temple.

**Objective:**

To assess the precise localization and volume of the hollowing defect, to optimize future cranioplasties.

**Methods:**

CT data of patients who received craniectomy and conventional CAD cranioplasty in our institution between 2012 and 2018 were analyzed. CT datasets prior to craniectomy and after cranioplasty were subtracted to quantify the volume and localization of the defect.

**Results:**

Out of 91 patients, 21 had suitable datasets. Five cases had good cosmetic results with no defect visible, 16 patients had an apparent hollowing defect. Their average defect volume was 5.0 cm^3^ ± 4.5 cm^3^. The defect localizations were in the area behind the zygomatic process and just below the superior temporal line, covering an area of app. 3x3 cm^2^. Surgical attempts of temporal muscle restoration were more often found in reports of good results (p<0.01), but also in 50% of reports, whose surgeries resulted in hollowing of the temple. Mean time between the two surgeries was 112 ± 43 days. No significant differences between patients with and without hollowing defect were detected regarding time between the two surgeries, age or performing surgeon.

**Conclusion:**

This work supplies evidence for the indication of a surgical corrective during cranioplasty in the small but cosmetically relevant area of the “frontozygomatic shadow”. Based on our 3D data analysis, future focused surgical strategies may obtain better aesthetical results here.

## Introduction

Decompressive craniectomy remains a debatable but without doubt life-saving surgery in otherwise uncontrollable intracranial hypertension [1–4]. Among the surviving patients, the majority will be scheduled for cranioplasty approximately 6–12 weeks later [5–7]. Following both procedures, craniectomy and cranioplasty, eventually a residual hollowing of the temple will occur in a number of cases due to inadequate positioning or volume of the temporal muscle. This can lead to an unsatisfying cosmetic result, which is especially important for those patients with good neurological outcome who are returning to social and professional life.

A number of surgical technical nuances both during craniectomy (e.g. limitation of monopolar cauterization, placement of layer-preserving material between dura and temporal muscle) and during cranioplasty (e.g. muscle dissection, muscle fixation to the implant) have been reported in the literature [1,8]. Nonetheless, it is our frequent observation that in spite of these efforts, residual hollowing of the temple occurs. From our surgical experience with the procedure the assumption arises that the measures mentioned above do not supply sufficient soft tissue substance preservation at all visible areas of the temple. Our hypothesis is that at the time of cranioplasty—additionally to the maximal restorable muscle substance and optimal muscle fixation—the area just behind the zygomatic process, from the superior temporal line downwards, requires further augmentation in many cases in order to avoid visible hollowing.

The aim of this work is to confirm this hypothesis analysing the frequency, precise localization and volumetric quantification of the hollowing defect after cranioplasty on the basis of 3D image analysis of a collective of patients with defects. We intend this work as foundation for a surgical augmentation technique of the expectable defect at the time of cranioplasty.

## Materials and methods

For this study, all institutional, national ethics committee and PLOS One Journal guidelines were followed. It was approved by the local ethics committee of the “Landesärztekammer Rheinland-Pfalz” (Reference: 2019–14726). Due to the fully retrospective and fully anonymized analysis the ethics committee waived the requirement for informed written consent of each individual. For publication of the illustrative photos of the case report the individual in this manuscript has given written informed consent (as outlined in PLOS consent form) to publish these case details.

All patients of our institution with operative procedural codes of craniectomy AND cranioplasty between January 2012 and December 2018 were searched in our institutional clinical information system. In all of these patients, the available imaging data stored in our PACS was screened. The imaging was suitable for analysis when 3D datasets (CT of 1mm or less slice thickness covering at least the whole surface of the temple) of both time points, prior to craniectomy (pre-craniectomy CT, ***pre-CT***) and more than 6 weeks after cranioplasty (late-post-cranioplasty CT, ***late-CT***), were available for the same patient. These minimum six weeks were chosen to insure that the final cranioplasty result at the temple without influence of postoperative swelling was seen.

We only used custom-shaped patient-specific implants for cranioplasty. We do not re-implant autologous bone flaps in our institution because of the evidence of increased complications regarding infection and bone resorption [9].

**Fig 1:**
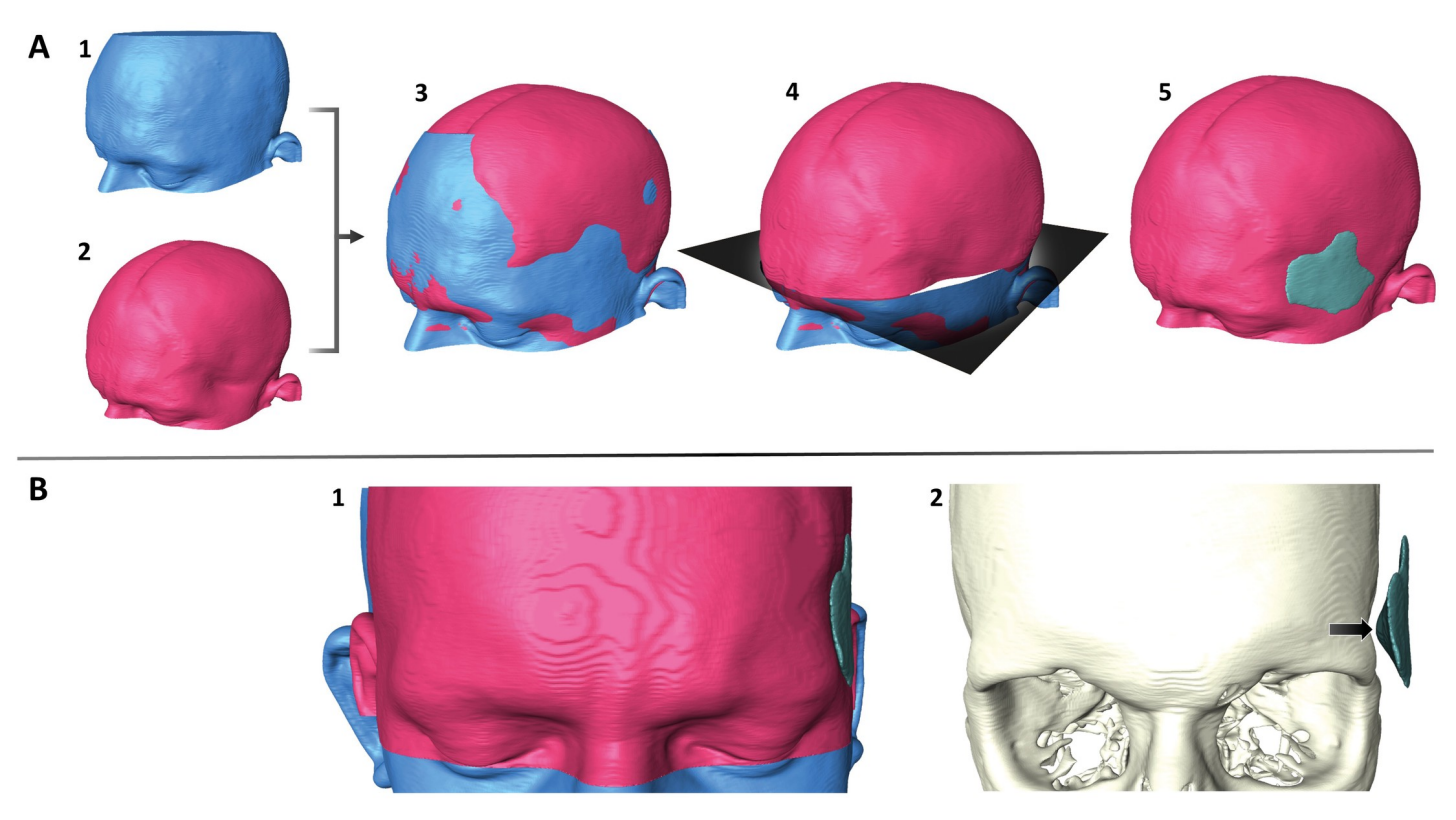
Illustration of the workflow of the 3D analysis at the example of one representative case. **A1** pre-CT before craniectomy, **A2** late-post-cranioplasty CT (late-CT) with clearly visible hollowing of the left temple, **A3** fusion of both datasets, **A4** resulting defect volume after subtraction of both datasets visualized as a cross section, **A5** protocol-optimized defect volume visualized as 3D surface object. **B1** front view of pre-CT and late-CT fused on each other with reconstructed defect volume on the left temple, **B2** front view of the defect volume in relation to the bone, **arrow:** Center of the defect volume.

In all of these cases, our 3D CT image analysis was performed with a scientific visualization software (AMIRA 5.4.5, Thermo Fisher Scientific, Massachusetts, USA) according to the following protocol:

1. Import and correction of CT data due to gantry tilt (Fig 1, A1 and A2).
2. Fusion of the late-CT dataset on to the pre-CT using a 6 degree of freedom registration module in Amira (Fig 1, A3).
3. Skin surface reconstruction in both datasets.
4. Subtraction of both surface datasets resulting in a defect volume dataset (Fig 1, A4).
5. Surface generation of the defect volume (Fig 1, A5).

Further software processing allowed to visualize the shape of the hollowing defect as a volume, to quantify its volume and to localize the center of the defect volume (defined as the thickest part of the volume) in reference to the skull (Fig 1).

**Fig 2:**
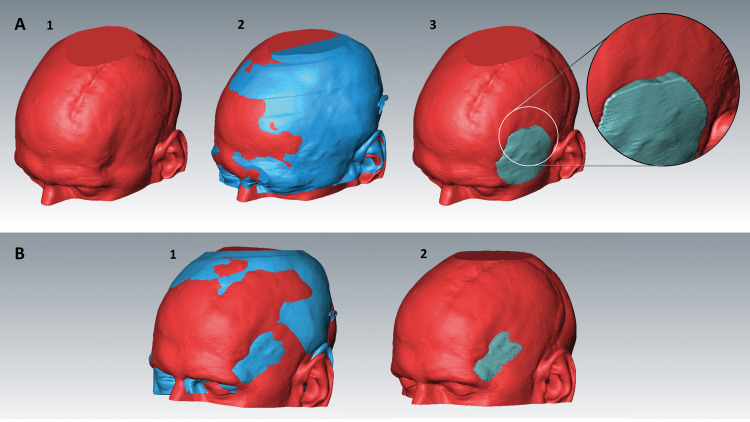
Illustration of manual skin surface matching in cases of insufficient automatic matching quality. **A1** late-CT with hollowing defect of the left temple, **A2** insufficient automatic matching due to soft tissue swelling in pre-CT (head trauma case, blue), **A3** inaccurate, wedge formed defect volume**. B1** manually corrected skin surface matching to obtain the narrowest and best isolated defect volume at the aesthetically relevant area **B2** resulting correct defect volume.

Although pre-CT and late-CT of the same patient will always match perfectly on the bone level, major mismatches of the skin surfaces before and after surgeries occur due to scalp scarring (Fig 2, A1 and A2). This results in meaningless hemispheric defect volumes (Fig 2, A3). In such cases, manual correction of the initial computer suggested fusion was performed to isolate and narrow the defect volume at the apparent esthetical skin surface defect (Fig 2, B1 and B2). All manual fusion corrections were performed by the same examiners in accordance (M.K. and M.J.). For further information see also S1 Appendix.

### Coloured

The 16 cases of the “hollowing group”. *Left in each*: surface reconstruction of late-CT with temple defect. *Right in each*: extracted 3D volume of defect in turquoise. The marginalia *auto* and *man*. indicate whether the matching succeeded by mere automatic computed matching of pre-CT and late-CT or required additional manual matching.

### Monochrome

The 9 cases rejected due to surface problems leading to meaningless analysis (n = 4) or because of absence of apparent temple defect (the “no defect” group, n = 5).

All individual defect volumes were matched to one reference head to obtain an averaged indicator for location and depth of the defect volume in relation to the skull bone. Therefore, all left sided defects were mirrored to the right side. Subsequently, all individual heads including their respective defect volumes were registered to the reference head by using the “affine registration” module of Amira again, but this time with 10 degrees of freedom. The resulting distorted dataset was accepted when the temporo-orbito-zygomatical bones showed a plausible alignment.

Next, all defect volumes were added together in one dataset to obtain a heat map showing the locations of all defect volumes in relation to the reference skull.

To measure the locations of the deepest point of the defect volumes in relation to the reference skull, the deepest point of the defect was marked in each volume (see arrow in Fig 1 B2) using the 3D software blender (version 2.82a, Stichting Blender Foundation, Amsterdam, Netherlands). The mean point was calculated and all points were projected on the reference skull from a lateral viewpoint.

The collective was then further subdivided for analysis of possible influencing parameters. Patients without apparent defect, i.e. with a good cosmetic result, were grouped together (no defect), and patients with an apparent hollowing defect of the temple were grouped together (hollowing). Different parameters between the groups were analysed (age, time between the two surgeries, surgeon and surgical technique (documented attempt of temporal muscle restoration during cranioplasty in the surgical report)) regarding their influence onto the defect volume. Statistical analysis was expressed in mean and standard deviation, and significance was assessed with the student´s t-test (Excel, Microsoft, California, USA).

## Results

The surgical codes linking craniectomy AND cranioplasty led to 91 patients in the mentioned time period. Of these, 25 patients had imaging datasets in our PACS available that allowed performing the analysis. The demographic and underlying pathology information is shown in Table 1.

**Table 1:** Demographic data of patients and diagnosis requiring their respective craniectomies.

**total number of searched patients**	**91**
** suitable imaging data available**	**25**
Excluded after 3D analysis	4
no defect (= good cosmetic result)	5
hollowing	16
** average age (n = 25)**	**52 ± 14**
** gender (n = 25)**	
female	10
male	15
** diagnosis (n = 25)**	
malignant ischemic stroke	9
hemorrhagic stroke	2
aneurysmatic SAH	2
AVM	1
trauma	11
** side (n = 25)**	
left	11
right	14

Of these 25 patients, 3D reconstructions showed a good cosmetic result without an apparent hollowing defect in 5 cases (24%, “**no defect” group, n = 5**), and 4 cases had to be excluded due to misfitting of cranioplasty implant or scalp scarring that resulted in meaningless 3D analysis. For the datasets of the remaining 16 patients who did have apparent hollowing of the temple (76%, **“hollowing” group, n = 16**), the analysis mentioned above was performed. The mean defect volume in the “hollowing” group was 5.0 ± 4.5 cm^3^ (min: 0.4 cm^3^, max: 16.7 cm^3^).

The mean time between the two surgeries was 112 ± 43 days (n = 25). The mean time between the two surgeries in the “no defect” group of 5 patients was 131 ±72 days. In the “hollowing” group of 16 patients, the mean time between the two surgeries was 108 ± 34 days. This difference was not statistically significant (p = 0.51).

The mean age in total (52 ± 14 years) and in the two subgroups (“no defect” group 53 ± 11 years; “hollowing” group 53 ± 16 years) did not differ significantly. Among the surgeons, no one with better results than others could be detected.

### Surgical temporal muscle restoration applied during cranioplasty

The surgical reports of all patients were read for descriptions of the respective temporal muscle restoration during cranioplasty. No muscle augmentation technique had been applied in any of the surgeries. In the “no defect” group, all 5 reports contained dedicated descriptions for the muscle restoration and attachment. In the “hollowing” group (n = 16), such dedicated descriptions were found for 8 patients. In 2 cases, dedicated descriptions of infeasibility to restore the muscle were found. In the remaining 6 cases, the description concerning the surgical management of the temporal muscle was either not clear or not mentioned. The difference between the two groups concerning this surgical report analysis was significant (p< 0.01).

### Localisation of the defect

**Fig 3:**
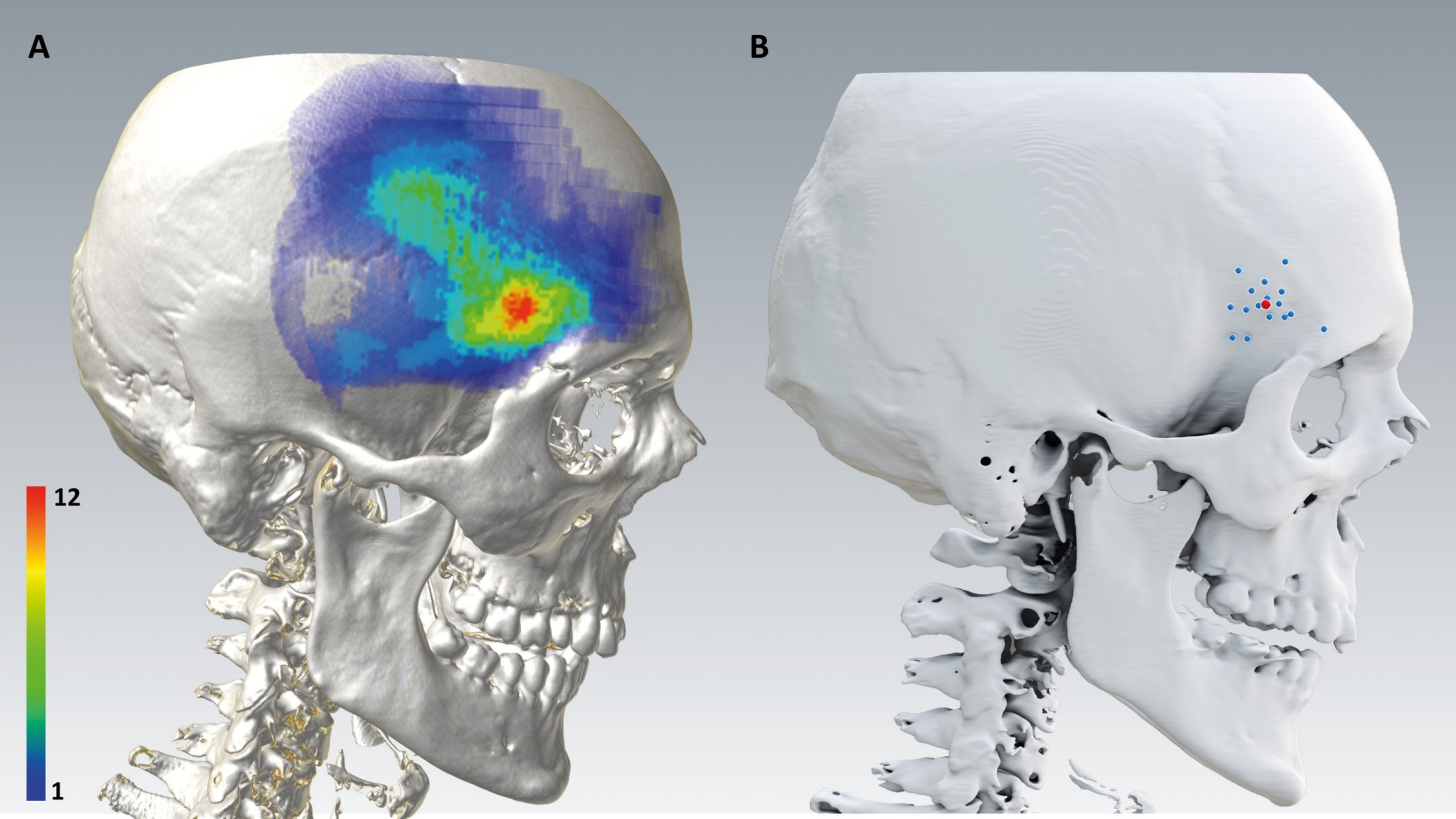
Matching of all 16 cases harbouring an apparent hollowing defect to one head of reference. **A:** Heat map depicting the areas with high intersections between the individual defects (blue: 1 case, red: 12 cases, total: 16 cases) **B:** Localisations of the deepest point of each single defect (blue) and their mean position (red) projected onto the skull bone from a lateral point of view.

All defect volumes were fused in one dataset to obtain a heat map depicting the area of the highest intersection over all defect volumes (Fig 3A). The localisation of the center of the defect volumes of all 16 accumulated cases is shown in Fig 3B.

## Discussion

The analyses revealed that despite the use of temporal muscle preserving techniques, approximately 2/3 of the patients show soft tissue defects of the temple.

**Fig 4:**
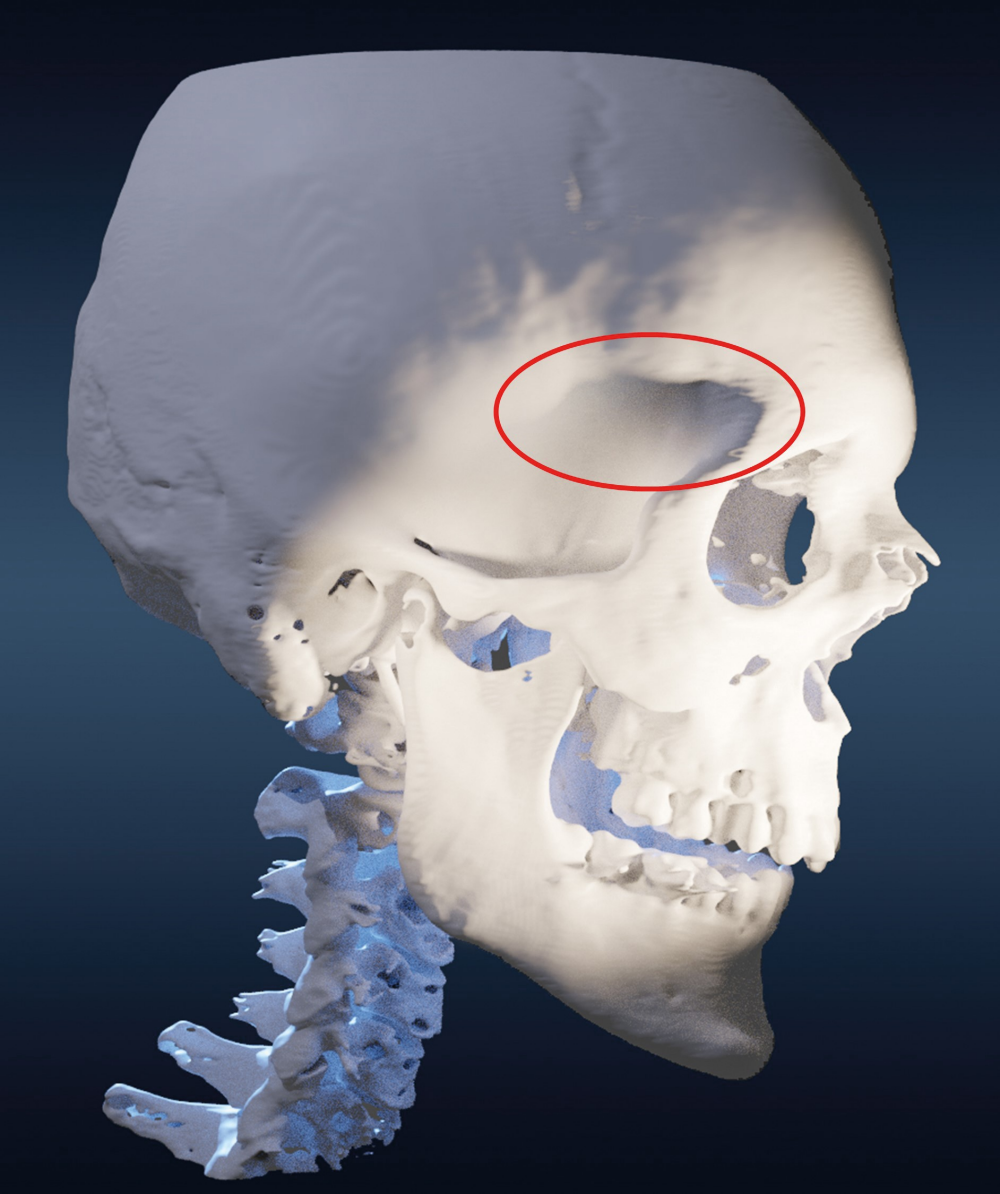
The frontozygomatic shadow. The shadow that is cast by the bony prominences of the superior temporal line and the zygomatic process of the frontal bone when illuminated from respective light source positions.

The motivation for this work comes from our intraoperative observation that in spite of adherence to published technical nuances of temporal muscle preservation and restoration, a region of relatively small volume but of esthetical importance–the approximately 3x3 cm area behind the zygomatic process of the frontal bone and below the superior temporal line–is not well enough addressed by these efforts in the majority of cases. For simplicity we will call this area **the frontozygomatic shadow** from here on (Fig 4).

The restored temporal muscle part cannot easily be attached high enough to the temporal line to restore the resulting natural contour of the temple in most cases. Hence, the muscle will have to be fixed at a lower level. Consequently, the orbital zygoma will appear more prominent under its fine skin, and the contour defect will become apparent. The limited quality of muscle fixation will often lead to a certain muscle bulge lower over the zygoma which further increases the optic impression of hollowing of the temple. We only used patient-specific CAD implants in this series that will–at the time of cranioplasty—cover all surgical bone defects including those resulting from burrholes or from drilling of the sphenoid wing at the time of craniectomy. We can therefore exclude that these play a major role for the development of a residual hollowing of the temple after cranioplasty and for our concept of the “frontozygomatic shadow”.

Many authors have addressed this problem with focus on the central temporal fossa, but do not point on this relatively small but aesthetically important volume defect at this high localization: First, temporal muscle damage during craniectomy. Namely, use of monopolar cauterization which can cause atrophy should be limited [1]. Second, due to the loss of function of the muscle in the period between craniectomy and cranioplasty muscle atrophy occurs. Third, the difficulty to dissect between the muscle and the dura mater during cranioplasty, with further damage to the remaining muscle. Several authors have suggested to place a dural substitute between dura and muscle after craniectomy [8]. Thus, at the time of cranioplasty, the plane between muscle and neo-dura should be easier to develop [8,10,11]. Honeybul et al. published a single cranioplasty case where the dura was opened and the temporal muscle left on it, and a dura substitute was used to close the dural defect [1].

At the time of cranioplasty, dissecting along the bone margins of the craniectomy at the temporal base helps to develop the correct plane between muscle and dura. The mobilized remaining muscle should then be fixed to the implanted prosthesis/bone flap with sutures or microplates. This muscle and thereby contour restoration can be further enforced by adding volume at the expected defect: Im et al. published a series of 99 patients who were treated at the time of pterional craniotomy with a polyethylene implant that was tailored to the defect during surgery and fixed with screws to the skull before closure [12]. Marbacher et al. describe a clinical case report of a patient who underwent replacement of an infected PMMA bone implant by a CAD PEEK prosthesis in which additional volume had been added by the manufacturer at the temple following the surgeon’s instructions [13] Park et al. performed a similar approach with a modified CAD implant with temporal enforcement in 10 patients and saw better esthetical outcomes than in 10 comparative patients who underwent conventional CAD prosthesis implantation [14]. This is today not possible by automated algorithms because of the CT density similarity of muscle, fat and soft tissue of the scalp, but has to be shaped individually in a fairly time-consuming manner–basically similar to ours in this study.

### Implication for surgical correction of the contour at time of cranioplasty

Obviously, this work is the prerequisite for a surgical correction of the expected hollowing at the time of cranioplasty. It was also our initial thought that the modification of the CAD prosthesis itself is the optimal strategy. Although comfortable at the time of surgery, this strategy has however several disadvantages. CAD implants are shaped from CT data according to the bone defect. However, it is arbitrary at the time of implant production to predict the future soft tissue volume defect—it is only seen at the time of cranioplasty, how much temporal muscle substance can be restored, up to what height it can be fixed to the prosthesis and hence to what extent a defect in the region of the frontozygomatic shadow will result. This favours an approach with an approved mouldable material for individual shaping during cranioplasty, eg. polymethylmethacrylate (PMMA). Another advantage of “adding substance” at this area is that it can be used for easy placement of fixation sutures of the muscle for a more effective suture fixation of the muscle.

### Comparison between patients with good and poor cosmetic results

There was a striking difference between the two groups concerning the mentioning of temporal muscle restoration in the surgical report analysis. In spite of the limited cases in the “no defect” group and the retrospective quality of this data, we believe that it indicates the importance of this surgical effort for the cosmetic result. In some cases, these conventional muscle restoration techniques are evidently sufficient to avoid hollowing of the temple. On the other hand, in 8 cases in the “hollowing” group, muscle restoration did not prevent evident hollowing. This underlines our concept of the frontozygomatic shadow as region of cosmetic importance. The other assessed parameters (time between surgeries, age and surgeon) did not influence the cosmetic result in our series.

### Image fusion of pre-CT and late-CT

The image fusion protocol applied in this study is examiner depending. Yet, we consider it the correct fundament for the consecutive volumetric analysis for the addressed issue. When we first analysed all cases by automated computed fusion of several mathematical algorithms (affine registration, 3-point-matching of prominent bone landmarks) the resulting defect volume was geometrically precise, but useless in the majority of cases in pointing at the esthetical problem, because minor differences between pre-CT and late-CT were apparent all over the hemisphere. This can be due to skin flap scarring, initial soft tissue swelling in the case of head trauma, change of body weight and other reasons. This observation motivated us to adjust the computed fusion manually to the impression of the examiner of the best-narrowed volume necessary to make the apparent defect disappear. The addressed issue of this work is of esthetical nature. The fusion protocol based on examiners´ eyes is therefore suitable.

The same applies for the matching of all analyzed datasets with reference to one head, which is not of millimetric precision but since distortion manoeuvres remain proportional over the whole surface of the processed structures, the resulting image is of value for the information of the relevant area of the defect.

### Limitations

The study has a relatively small sample size and is of retrospective character. The lack of detailed descriptive information about the applied surgical techniques of muscle restoration is a consequence of this.

### Outlook

**Fig 5:**
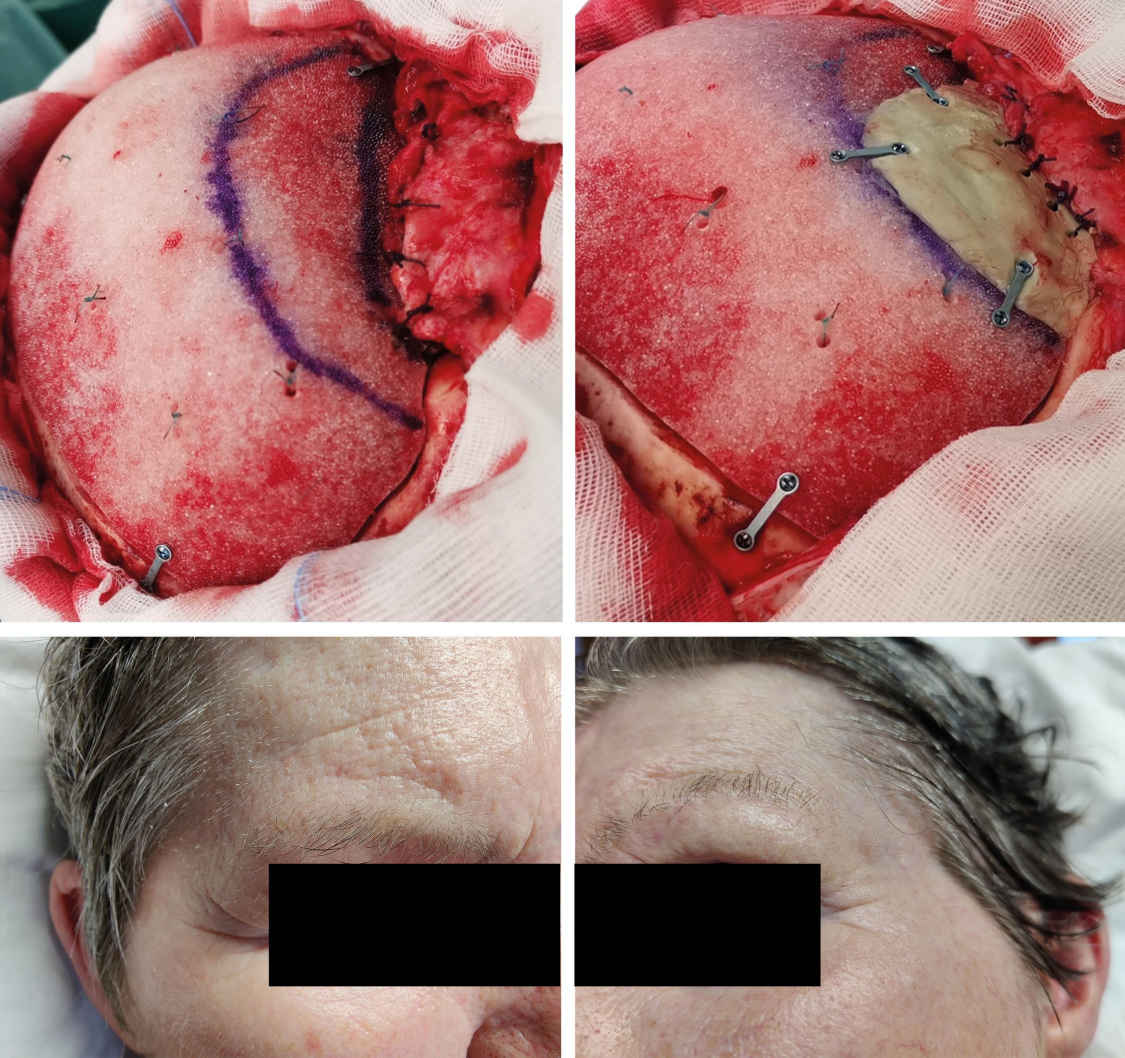
Photo illustration of a procedure of cranioplasty in which augmentation of the discussed region with PMMA was practised, and of the cosmetic result 1 year after surgery. **Top left:** Defect at the discussed region. Lower blue pen mark: Highest possible position of fixation of the restored temporal muscle. Higher blue pen mark in correspondence to the superior temporal line. **Top right:** Correction of the expected defect for the region of the frontozygomatic shadow with a PMMA bone cement substitute added to the CAD prosthesis. **Bottom line:** Photo of the patient 1 year after cranioplasty performed on the left patient side. Note that this patient naturally has a prominent zygoma on both sides, but that there is a good cosmetic result with a symmetric frontozygomtic shadow. (publication with kind authorisation of the patient/next of kin).

In a recent case at our institution, we were faced by an unusually insufficient temporal muscle restoration during cranioplasty. The cosmetic result had to be expected to be not acceptable for the patient. Hence, the surgical team decided to reduce the defect by PMMA bone cement as approved material. Fig 5 shows the result 1 year after cranioplasty (the patient/next of kin consented to publication of her image).

## Conclusions

The hollowing defect of the temple following cranioplasty is of relatively small volume, but at an esthetically critical area, where conventional surgical techniques often fail to restore soft tissue volume. This work supplies evidence for the indication of a surgical corrective at the region of the frontozygomatic shadow at the time of cranioplasty.

## Supporting information

S1 Appendix Illustration of all 25 individual 3D analyses performed. (TIF)

S1 Data (XLS)

## References

[1] Honeybul S. Management of the temporal muscle during cranioplasty: technical note. Journal of neurosurgery Pediatrics. 2016;17(6):701–4. Epub 2016/02/20. doi: 10.3171/2015.11.PEDS15556 . 26894519

[2] Hutchinson PJ, Kolias AG, Timofeev IS, Corteen EA, Czosnyka M, Timothy J, et al. Trial of Decompressive Craniectomy for Traumatic Intracranial Hypertension. The New England journal of medicine. 2016;375(12):1119–30. Epub 2016/09/08. doi: 10.1056/NEJMoa1605215 . 27602507

[3] Jüttler E, Schwab S, Schmiedek P, Unterberg A, Hennerici M, Woitzik J, et al. Decompressive Surgery for the Treatment of Malignant Infarction of the Middle Cerebral Artery (DESTINY): a randomized, controlled trial. Stroke. 2007;38(9):2518–25. Epub 2007/08/11. doi: 10.1161/STROKEAHA.107.485649 . 17690310

[4] Beck J, Fischer U. The SWITCH trial—Swiss Trial of Decompressive Craniectomy versus Best Medical Treatment of Spontaneous Supratentorial Intracerebral Hemorrhage: A Randomized Controlled Trial. 2020 [cited 2020 Nov. 5, 2020]. Available from: https://www.switch-trial.ch/.

[5] Cho YJ, Kang SH. Review of Cranioplasty after Decompressive Craniectomy. Korean journal of neurotrauma. 2017;13(1):9–14. Epub 2017/05/18. doi: 10.13004/kjnt.2017.13.1.9 ; PubMed Central PMCID: PMC5432454. 28512612 PMC5432454

[6] Ashayeri K, E MJ, Huang J, Brem H, Gordon CR. Syndrome of the Trephined: A Systematic Review. Neurosurgery. 2016;79(4):525–34. Epub 2016/08/05. doi: 10.1227/NEU.0000000000001366 . 27489166

[7] Annan M, De Toffol B, Hommet C, Mondon K. Sinking skin flap syndrome (or Syndrome of the trephined): A review. British journal of neurosurgery. 2015;29(3):314–8. Epub 2015/02/28. doi: 10.3109/02688697.2015.1012047 . 25721035

[8] Missori P, Polli FM, Peschillo S, D’Avella E, Paolini S, Miscusi M. Double dural patch in decompressive craniectomy to preserve the temporal muscle: technical note. Surgical neurology. 2008;70(4):437–9; discussion 9. Epub 2007/08/21. doi: 10.1016/j.surneu.2007.03.029 . 17707489

[9] Liu L, Lu ST, Liu AH, Hou WB, Cao WR, Zhou C, et al. Comparison of complications in cranioplasty with various materials: a systematic review and meta-analysis. British journal of neurosurgery. 2020;34(4):388–96. Epub 2020/04/03. doi: 10.1080/02688697.2020.1742291 . 32233810

[10] Bulters D, Belli A. Placement of silicone sheeting at decompressive craniectomy to prevent adhesions at cranioplasty. Br J Neurosurg. 2010;24(1):75–6. doi: 10.3109/02688690903506135 . 20158357

[11] Miyake S, Fujita A, Aihara H, Kohmura E. New technique for decompressive duraplasty using expanded polytetrafluoroethylene dura substitute—technical note. Neurol Med Chir (Tokyo). 2006;46(2):104–6; discussion 6. doi: 10.2176/nmc.46.104 . 16498223

[12] Im SH, Song J, Park SK, Rha EY, Han YM. Cosmetic Reconstruction of Frontotemporal Depression Using Polyethylene Implant after Pterional Craniotomy. Biomed Res Int. 2018;2018:1982726. doi: 10.1155/2018/1982726 ; PubMed Central PMCID: PMC6215591. 30420957 PMC6215591

[13] Marbacher S, Andereggen L, Fandino J, Lukes A. Combined bone and soft-tissue augmentation surgery in temporo-orbital contour reconstruction. J Craniofac Surg. 2011;22(1):266–8. doi: 10.1097/SCS.0b013e3181f7b781 . 21233740

[14] Park SE, Park EK, Shim KW, Kim DS. Modified Cranioplasty Technique Using 3-Dimensional Printed Implants in Preventing Temporalis Muscle Hollowing. World Neurosurg. 2019;126:e1160–e8. doi: 10.1016/j.wneu.2019.02.221 . 30880206

