# Supplementary figures and images for: Quantitative assessment and localization of the hollowing of the temple after craniectomy and cranioplasty–The frontozygomatic shadow
Source: PLoS One. 2021 Oct 19;16(10):e0258776. doi: 10.1371/journal.pone.0258776 (PMC8525753; doi:10.1371/journal.pone.0258776)

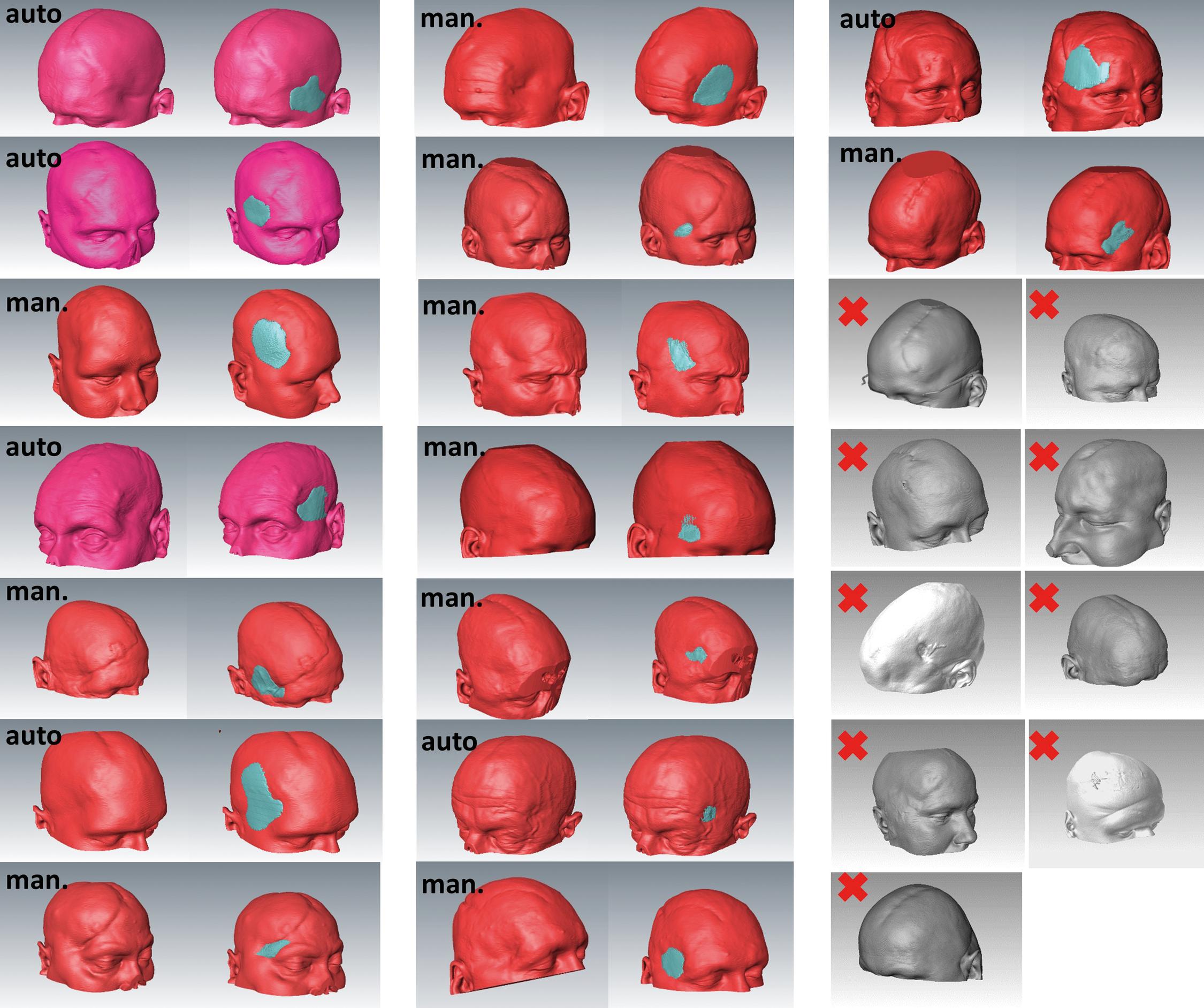

Supplement: S1 Appendix — (TIF) [file pone.0258776.s001.tif]
